# Improved estimation of structure predictor quality

**DOI:** 10.1186/1472-6807-9-41

**Published:** 2009-06-30

**Authors:** Kevin W DeRonne, George Karypis

**Affiliations:** 1 Department of Computer Science & Engineering, Digital Technology Center University of Minnesota, Minneapolis, MN 55455, USA

## Abstract

**Background:**

Methods that can automatically assess the quality of computationally predicted protein structures are important, as they enable the selection of the most accurate structure from an ensemble of predictions. Assessment methods that determine the quality of a predicted structure by comparing it against the various structures predicted by different servers have been shown to outperform approaches that rely on the intrinsic characteristics of the structure itself.

**Results:**

We examined techniques to estimate the quality of a predicted protein structure based on prediction consensus. LGA is used to align the structure in question to the structures for the same protein predicted by different servers. We examine both static (e.g. averaging) and dynamic (e.g. support vector machine) methods for aggregating these distances on two datasets.

**Conclusion:**

We find that a constrained regression approach shows consistently good performance. Although it is not always the absolute best performing scheme, it is always performs on par with the best schemes across multiple datasets. The work presented here provides the basis for the construction of a regression model trained on data from existing structure prediction servers.

## Background

The problem of predicting protein structure from amino acid sequence has yet to be fully solved, and experimentally determining protein structures requires extensive human input. Due to the relative ease of determining amino acid sequences, and the utility of structural information, the problem has attracted much attention. As the accuracy of protein structure prediction algorithms has greatly improved over the last ten years [1,2], the ability to precisely determine the quality of protein structure predictions has gained importance. In an attempt to motivate improvements in this area, the most recent session of the Critical Assessment of Structure Prediction (CASP) included a model quality assessment category [3]. CASP is A biennial competition  that has been organized to motivate advancements in the area of protein structure prediction. For this new category, given a putative structure, competitors were asked to submit a quality score between 0.0 and 1.0, or to assign an error estimate (in Å) to each residue of the structure. Twenty-eight groups submitted full structure quality estimates, and eight of those submitted per-residue error estimates.

These eight groups assess structure quality using a variety of methods, which can be broadly grouped into four classes. The first class of techniques learns models to assess the per-residue error based on intrinsic properties of the predicted structures. The second class forms a prediction based on the quality of a target-template alignment. The third class determines the error by taking into account different per-residue error predictors. Finally, the fourth class relies on the consensus of the predictions obtained from different protein structure prediction servers. Techniques in the first class include ProQ [4], Victor/FRST [5] and QUAD [6]. ProQ uses neural networks to learn a model based on atom-atom contacts, residue-reside contacts, and agreement with predicted secondary structure. Victor/FRST uses a linear combination of four potential energy functions: a pairwise potential, a solvation potential, a torsion angle potential and a hydrogen bond potential. The relative weights for each function are optimized on the CASP4 decoy set. QUAD combines secondary structure, hydrogen bonding, and solvent accessibility information to produce a fitness score for each residue in its structural environment. Techniques in the second class include ProQProf [7] and SUBWAy [8]. ProQProf trains a neural network on profile-profile comparisons from pairs of profiles for target/template sets. SUBWAy generates multiple suboptimal alignments to a template for a target sequence. The average deviation between the suboptimal alignments and the optimal alignment serves as an indicator of structure quality. (A higher average deviation indicates a lower quality structure.) Techniques in the third class include ProQlocal [7] and Meta-MQAP [9]. A prediction by ProQlocal is a sum of the scores from ProQProf and ProQ. Meta-MQAP queries other quality assessment servers and combines the results. Lastly, Pcons [10] makes up the fourth class, which is based on the idea that within an ensemble of structures predicted from different servers for the target protein, recurring structures and structural motifs have an increased probability of being high-quality (i.e., close to the native state). Driven by this principle, Pcons determines the quality of a structure in two steps. First, it performs pairwise LGscore alignments [11] between the query structure and all structures in the ensemble, and second it uses the average *S*-scores [12] computed from these alignments to determine the per-residue error predictions. The results of the CASP7 competition showed that Pcons was the most successful approach in predicting both complete structure quality and per-residue errors and it significantly outperformed the next best scheme.

In this paper we focus on improving the per-residue error estimates using consensus-based methods. First, we examine several static methods for consensus prediction. Second, we look at whether different predictors provide enough consistency for machine learning models to be effective. In doing so, we present the use of a linear perceptron, standard regression, support vector regression and a simple weight learning technique to learn an appropriate aggregation scheme. Extensive experimental evaluation on data obtained from the CASP6 and CASP7 competitions demonstrates that constrained regression provides the biggest gains in performance over the previous best performing scheme (Pcons). The results of these experiments indicate that an improved model quality assessment server could be built. This server would rely on a series of predictions from existing assessment servers in order to build a constrained regression model. Given a new structure for assessment, the server would first query these servers. Then, it would apply the constrained regression model to produce the final quality assessment.

## Results and discussion

### Static Consensus Error Prediction Methods

The work in this paper builds upon a consensus-based per-residue error prediction method rooted on the same principles introduced by Pcons. The general procedure works as follows. Let *X *be the amino acid sequence of the query protein and let *S*_*X *_be its predicted 3D structure. Let  be the true 3D structure for *X *and let  be the structures of *X *predicted by *k *different structure prediction methods. For each residue *x*_*i *_of *X*, let *d*_*j*_(*x*_*i*_) be the distance between the *i*th residue of *S*_*X *_and the *i*th residue of  obtained after structurally superimposing *S*_*X *_and  using the LGA program [13]. The predicted distance *d*(*x*_*i*_) between residue *x*_*i *_in *S*_*X *_and  (the error estimate for position *i*) is given by

(1)

where *w*_*j *_is a weight associated with the *j*th predictor and ∑_*j*_*w*_*j *_= 1.0. The idea behind this approach, which is shared by Pcons, is that each of the *k *structures over which it averages can be treated as an expert's prediction for *X*'s real structure. Thus, the per-residue error can be determined by a weighted average over the per-residue distances to the structure of each expert. The various *w*_*j *_parameters control how the distances between the query and the predictors are weighted. A straightforward approach is to treat each of the predictors equally by making all these weights equal (i.e., *w*_*j *_= 1/*k*), which corresponds to simple averaging.

The above method differs from Pcons in two important ways. First, it uses LGA alignments as opposed to LGscore alignments. An LGA alignment is constructed by attempting to optimize both the longest continuous sequence that can fit under a certain cutoff, as well as a global measure of alignment quality. An LGscore alignment attempts to find the most significant [12] non-continuous segment of a model. Second, it averages the raw distances as opposed to *S*-scores [11]. *S*-scores were developed as part of an improvement over root mean square deviation calculations for global structural comparison [12] (see the Methods section for how the *S*-score is calculated).

To understand the impact of these choices, we examine the performance obtained by four different schemes. The first two schemes (LGA-Distance and LGA-*S*-score) use LGA to compute alignments, and average either raw distances or *S*-scores, whereas the second two schemes (LGscore-Distance and LGscore-*S*-score) use LGscore to compute alignments, again averaging either raw distances or *S*-scores. Using LGscore alignments and averaging *S*-scores is identical to the Pcons approach, and so we equivalently refer to Pcons as the LGscore-*S*-score scheme. The prediction performance achieved by these methods on two datasets obtained from the CASP6 and CASP7 competitions, (labeled CD6 and CD7, respectively), is shown in Table 1. The performance was assessed by calculating both the Pearson correlation coefficient (CC) and the root mean squared error (RMSE) between the actual and predicted per-residue errors.

**Table 1:** Prediction performance of the static methods

	CD6		CD7	
Method	CC	RMSE	CC	RMSE

LGA-Distance	0.68	11.71	**0.79**	**7.49**
LGA-*S*-score	0.66	**11.06**	0.74	7.78
LGscore-Distance	0.66	11.46	0.77	7.66
LGscore-*S*-score	**0.70**	11.19	0.76	8.18

Values in this table represent the Pearson correlation coefficient (CC) and root mean squared error (RMSE) between the predicted and true per-residue distances. Boldfaced entries correspond to the best performing scheme for each dataset and performance assessment metric.

These results show that the performance of the various schemes differs across the two datasets. For CD6, LGscore-*S*-score achieves the best CC while LGA-*S*-score obtains the lowest RMSE. For the CD7 dataset, LGA-Distance shows the best performance in terms of CC and RMSE. The improvements achieved by the best performing schemes on the two datasets and metrics when compared to the next best schemes are significant at *p *≤ 0.0001. Comparing the performance of the schemes using distance-based averaging over those that average *S*-scores, we see that the former lead to better results on CD7, whereas their relative performance on CD6 is mixed. A similar dataset specific behavior can be observed by comparing the performance achieved by the two alignment methods. LGA-based alignments perform consistently better on CD7 whereas the relative performance of LGA- and LGscore-based alignments on CD6 changes based on the averaging scheme (distances or *S*-scores) and performance metric.

### Learning-based Consensus Error Prediction Methods

Motivated by the inconsistent performance achieved by the four consensus-based per-residue error prediction schemes discussed in the previous section, we investigate the extent to which we can obtain a consensus prediction method that achieves both a consistent and better performance across different datasets and performance assessment metrics. Toward this goal we focus on techniques that instead of treating each of the predictors equally by statically setting the *w*_*j *_parameters to 1/*k*, they use machine learning methods to estimate the various *w*_*j *_parameters directly from the data. We formulate the problem of learning the *w*_*j *_weights as the following supervised learning problem. Given a set of training examples *x*_*i*_, each described by the tuple (*d*_*t*_(*x*_*i*_), ⟨*d*_1_(*x*_*i*_), *d*_2_(*x*_*i*_),..., *d*_*k*_(*x*_*i*_)⟩), where *d*_*t*_(*x*_*i*_) is the actual distance between the *i*th residue of *S*_*X *_and the *i*th residue of *X*'s true structure, learn the set of *w*_*j *_values such that

(2)

is minimized.

Four different schemes were used to learn these weights: support vector regression (SVR), linear percep-tron, least-squares regression, and a variant of the least-squares regression that constraints the *w*_*j *_weights to be non-negative. Note that with the exception of the linear perceptron, the other three learning methods do not enforce the constraint that ∑_*j*_*w*_*j *_= 1.0. Most of the learning schemes (Table 2) outperform the static prediction methods (Table 1) on both the CD6 and CD7 datasets. The overall best results for both datasets were obtained by the constrained regression method, which achieved an improvement in terms of RMSE of 20.2% and 21.0% over the best performing static schemes for CD6 (LGA-*S*-score) and CD7 (LGA-Distance), respectively. Its performance in terms of CC was similar to that of the best static scheme for CD6 and better by 3.4% for CD7. Note that the relatively higher gains in terms of RMSE when compared to the improvements achieved in terms of CC is due to the fact that the objective function of the learning methods (Equation 2) is designed to directly minimize the RMSE of the predictions. Overall, the prediction improvements and consistency achieved by the four learning schemes and constrained regression in particular show that learning methods can be used to learn how to weight the predictions of each server in order to obtained better per-residue error estimates.

**Table 2:** Prediction performance of the weight-learning methods

	CD6		CD7	
Method	CC	RMSE	CC	RMSE

Support Vector Regression	0.68	10.35	0.80	6.41
Linear Perceptron	0.69	9.74	0.80	6.46
Standard Regression	**0.70**	9.32	0.80	6.28
Constrained Regression	**0.70**	**9.20**	**0.81**	**6.19**

Values in this table represent the Pearson correlation coefficient (CC) and root mean squared error (RMSE) between the predicted and true per-residue distances. Boldfaced entries correspond to the best performing scheme for each dataset and performance assessment metric.

**Figure 1:**
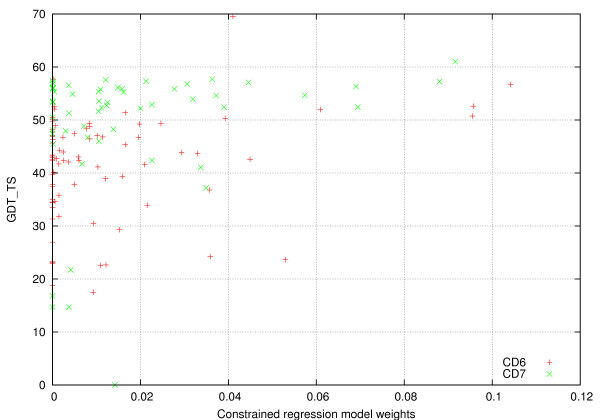
**Model weights vs Server quality**.

Since the nature of the model learned by the above methods is that of weighting the distances of the predictions obtained from each server, an important question to answer is how these weights are related to the overall quality of the predictions produced by each server. Figure 1 plots the weights learned by the constrained regression method against the quality of the structures generated by each server. The quality of a predicted structure was measured by its GDT_TS score. The values for the model weights and server GDT_TS scores correspond to the averages over the 80 and 58 structures used in the CD6 and CD7 datasets, respectively. From these plots we can see that even though there is a correlation between the weights and the GDT_TS scores (0.24 for CD6 and 0.22 for CD7), this correlation is not perfect. There are high-quality servers that are assigned low weights as part of the training, indicating that the information provided by them is redundant for estimating the per residue error. On the other hand, there are servers that do not perform extremely well, but they are still utilized by the learned model, suggesting that they provide key information for improving the error prediction.

#### Eliminating missing predictions

A potential issue with the above problem formulation is that, due to the way the training set is constructed, it may contain missing values. The missing values arise when a predictor could not provide a prediction for an entire protein, or just some of its positions. These missing values are assigned a value of zero, which may confuse the learning algorithms, as a zero value can mean two different things depending on its source.

If the original *d*_*j*_(*x*_*i*_) value is just missing, then a zero is uninformative. However, if the zero represents a true *d*_*j*_(*x*_*i*_) value of zero then the distance between *S*_*X *_and  at position *i *is zero. This means that the two structures align perfectly at this position. In this case *d*_*j*_(*x*_*i*_) should be treated as a zero, but in the former case *d*_*j*_(*x*_*i*_) should simply be ignored. In order to address this problem we developed two methods for adjusting the training data in order to compensate for the issue of *d*_*j*_(*x*_*i*_) values of zero.

The first method eliminates the confusion due to missing values by simply filling the missing values using estimates obtained from the other examples in the training set. (See the section called "Filling in missing values" in the Methods section for a precise description of how this is done.) The results achieved by this approach (Table 3) show that there is no clear advantage in building models on the filled datasets. Excluding the linear perceptron, which shows a markable decrease in RMSE performance, the gains achieved by the other methods are limited.

**Table 3:** Prediction performance of the weight-learning methods with filled values.

	CD6		CD7	
Method	CC	RMSE	CC	RMSE

Support Vector Regression	0.69	10.21	0.80	6.36
Linear Perceptron	0.68	11.31	0.79	7.45
Standard Regression	0.69	**9.48**	0.80	**6.26**
Constrained Regression	**0.71**	10.04	**0.81**	6.37

Values in this table represent the Pearson correlation coefficient (CC) and root mean squared error (RMSE) between the predicted and true per-residue distances. Boldfaced entries correspond to the best performing scheme for each dataset and performance assessment metric.

The second method addresses the missing values problem by creating a custom training set for each test position encountered. Specifically, for a given test position, the training set contains only those examples that have at least the same set of predictors present as those in the test position. As the set of positions tested by the custom training set approach are a subset of the CD6 and CD7 datasets, we refer to them as the PD6 and PD7 datasets, respectively. Exactly how these datasets differ from the CD6 and CD7 sets is described in the section called "Evaluating weight learning algorithms". Values for *w*_*j *_are learned from the training set, and these weights are used to classify the query position *q *according to Equation 1. Table 4 shows the results from testing models built using such custom data sets.

**Table 4:** The average correlation coefficient (CC) and RMSE for the PD6 and PD7 datasets.

	PD6		PD7	
Method	CC	RMSE	CC	RMSE

Custom training				
Support Vector Regression	**0.87**	**3.33**	**0.89**	**2.86**
Linear Perceptron	**0.87**	3.81	**0.89**	2.93
Standard Regression	0.70	6.02	0.82	3.80
Constrained Regression	**0.87**	3.46	**0.89**	**2.86**
Global training				
Support Vector Regression	**0.90**	**2.97**	**0.90**	**2.67**
Linear Perceptron	0.88	3.64	0.89	2.90
Standard Regression	0.89	3.20	0.90	2.76
Constrained Regression	0.89	3.15	0.89	2.79
Static consensus				
LGA-Distance	**0.86**	4.29	**0.89**	**3.19**
LGA-*S*-score	**0.86**	**3.74**	0.83	3.56
LGscore-Distance	0.67	5.56	0.72	4.48
LGscore-*S*-score	0.81	4.57	0.79	4.33

Values in this table represent the Pearson correlation coefficient (CC) and root mean squared error (RMSE) between the predicted and true per-residue distances. Boldfaced entries correspond to the best performing scheme for each dataset and performance assessment metric.

Because these results were obtained on the PD6 and PD7 datasets they are not directly comparable to those reported in Tables 2–3, which were obtained on the CD6 and CD7 datasets. For this reason, Table 4 also shows the performance of the weight-learning methods trained on the entire training set and the static consensus error prediction methods. These results are shown under the headings "Global training" and "Static consensus", respectively. Analyzing the performance of the custom training methods we observe that constrained regression once again shows consistently good performance, with the best correlation coefficient for both PD6 and PD7. Constrained regression also shows the best RMSE for PD7, and is within 4% of the best RMSE (3.46 as compared to SVR at 3.33) for PD6. However, comparing the results obtained by the custom training methods over those obtained by the methods trained using the entire training set (even when there are missing values), we see that the latter achieve consistently better performance. An explanation for this performance degradation is that the size of the training set of each custom training problem is relatively small, and as such the models are not as effective as those learned on the entire dataset.

## Conclusion

The results presented in this study reveal several interesting trends. First, a machine learning framework can be utilized to learn models that intelligently weight the different servers in the context of consensus-based error prediction. Second, constrained regression outperforms or performs on par with more complicated learning techniques while preventing over-fitting. This performance is consistent across two CASP datasets, in which care was taken to include as much data as possible. Third, filling missing values with an approximation does not produce consistent gains in performance. In the same vein, customizing training sets to assist machine learning techniques does not produce substantial gains in performance over static prediction methods on the same data. Moreover, their overall performance is considerably worse than that obtained by the models trained using all the training data, even when they contain missing values.

The types of models studied in this paper learn how to weight the predictions of a fixed set of protein structure prediction methods. Consequently, in order to use these models for assessing the quality of a protein's predicted 3D structure, the protein needs to also be predicted by the same set of prediction methods on which the model was trained. In our study, the training and testing of these models are done in the context of data obtained in the course of a CASP competition, satisfying the above requirement. However, models of this type can also be trained and applied in order to assess the quality of a protein's predicted 3D structure outside the context of a CASP competition. Specifically, a set of existing publicly available structure prediction servers will be used to define the *fixed *set of protein structure prediction methods whose weights will be learned by the models. By querying this set of servers with sequences from recently released structures, the resulting predictions can be used for training. During error prediction, the same set of servers will be used to predict the structure of the query protein *X *and their pairwise LGA alignment with *S*_*X *_will provide the *d*_*j*_(*x*_*i*_) distances in Equation 1 in order to compute the per-residue error estimates along with the weights learned during training. Moreover, as new prediction servers become available and old ones drop out of favor, the underlying models can be retrained to use the new set of prediction methods. Note that in the above framework, by restricting the training set to sequences that have only been recently structurally characterized ensures that the prediction servers actually make predictions, rather than just querying a structure database for the true structure. Finally, as the results in Figure 1 indicate, for many servers, the weights that are learned are either zero or very small, suggesting that the above framework can achieve good performance with a relatively small number of servers.

## Methods

### Dataset

The data used in this study comes from the CASP6 and CASP7 experiments. There are 66 target proteins and 57 predictors from CASP6, and 95 target proteins and 80 predictors from CASP7.

The LGA program [13] is used to align each pair of structures with the options -3 -ie -d:4.0 -o0 -sda. These are the same options used by the CASP assessors.

**Figure 2:**
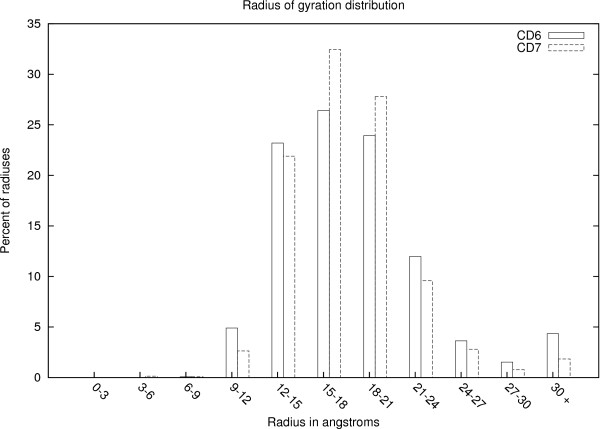
**Radius of gyration distribution**.

**Figure 3:**
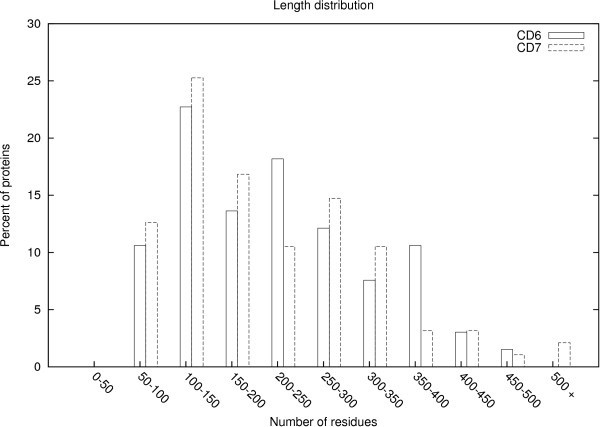
**Size distribution**.

Each of the CASP datasets can be viewed as a matrix, where the columns correspond to the different predictors, and the rows of the matrix are obtained by concatenating the sets of predictions from each of the residues. An (*i*, *j*) entry in the matrix represents the distance between a residue of the row submission to the corresponding residue in the column submission. These distances are derived after super-imposing the structures of the two submissions using LGA. If a particular predictor *j *did not submit a prediction for a protein *X*, the (*i*, *j*) entries for all rows *i *corresponding to protein *X *will be empty. Also, some (*i*, *j*) entries can be missing because the predictor did not provide predictions for these positions. We will refer to the matrices for the CASP6 and CASP7 datasets individually as the CD6 and CD7 matrices, respectively. The CD6 matrix contains data for 824,145 positions and the CD7 matrix contains data for 918,467 positions. Figure 2 shows a histogram of the radius of gyration values for the proteins in both the CD6 and CD7 datasets, while Figure 3 shows a histogram of pairwise sequence similarities. Radius of gyration is a measure of the structural size of a protein. As Figure 2 shows, the proteins in our datasets have a range of sizes. The proteins in both sets are spread across a wide variety of folds, with 33 unique folds in the CD6 set, and 37 unique folds in the CD7 set. For the CD6 set, the minimum occupancy for a fold was 1, the maximum was 4, and the average was 1.3. For the CD7 set, the minimum occupancy was 1, the maximum was 15, and the average was 2.1.

### Evaluating weight learning algorithms

We evaluate the three data-based approaches for learning the weights described in this paper (unfilled, filled, and custom training sets) using the CASP6 and CASP7 datasets as follows.

The unfilled and filled approaches are evaluated using a leave-one-protein-out framework. A protein is selected, and all positions from this protein are assigned to the test set. All remaining examples are used as the training set. The whole process is repeated for each available protein, so every position in the CD6 and CD7 matricies is used as a test position at some point. The difference between the unfilled and the filled approaches is that in the latter, the empty entries of the CD6 and CD7 matrices are filled using the method described in the next section.

The custom training set approach is evaluated under a leave-one-position-out framework. A single position *x*_*i *_serves as the test set and all positions from proteins other than *X *are searched to find the training set. The training set for position *x*_*i *_corresponds to those rows of the CD6 and CD7 matrices whose non-empty columns are a superset of the non-empty columns of *x*_*i*_'s row. Only the values from the set of non-empty columns in *x*_*i *_are used when training. This creates a training set with no missing values (i.e., the sub-matrix formed by the rows corresponding to the training set and the set of columns in the test position is completely filled). Note that it would seem that this approach will require learning 824,145 models for CASP6 and 918,467 models for CASP7 (one for each row of the CD6 and CD7 matrices). However, since the non-empty columns for the rows of the same protein will usually be the same (leading to the same training sets), the number of models actually required to be learned is much smaller. Occasionally, rows from the same protein will differ, due to incomplete CASP7 predictions, but these cases are rare. To further reduce the necessary number of models, we did not test those positions whose training sets could not be used for at least nine other positions (i.e., each model that we learned had to be used to test at least ten positions). This reduced the total number of models learned to only 553 for CASP6 and 1064 for CASP7, allowing us to test 96,089 positions for CASP6 and 198,612 positions for CASP7. We will refer to these datasets as the PD6 and PD7 datasets, respectively.

### Filling in missing values

The scheme that we used to fill in the missing values in the CD6 and CD7 matrices is based on similar techniques that were developed in the field of collaborative filtering [14-16]. An empty (*i*, *j*) position is filled by assigning to it a value that is obtained by averaging over the non-empty positions of column *j*, while taking into account the values along the rows in which these non-empty positions occur. Specifically, let *D *be one of the CD6 and CD7 matrices, let *μ*_*i *_= (∑_*j*_*D*(*i*, *j*))/*m*_*i *_be the mean value of the *m*_*i *_non-zero entries of row *i *in *D*, let *D' *be the matrix obtained from *D *by subtracting from each non-empty (*i*, *j*) position its row average (i.e, *D'*(*i*, *j*) = *D*(*i*, *j*) - *μ*_*i*_), and let *μ*_*j *_= (∑_*i*_*D'*(*i*, *j*))/*m*_*j *_be the mean value of the *m*_*i *_non-zero entries of column *j *in *D'*. Then, an empty position (*i*, *j*) is assigned the value *D*(*i*, *j*) = *μ*_*i *_+ *μ*_*j*_. Note that, in the case of filling a testing set, the values for *μ*_*j *_from the training set are used to fill any missing values.

The advantage of this method is that it accounts for differences in the underlying structural alignments, which is important because each of the alignments between a row prediction and a column prediction provides its own context. Subtracting *μ*_*i *_from each row places all of the rows into a generalized context, and allows for the accurate computation of *μ*_*j *_values. By subsequently adding *μ*_*i *_to *μ*_*j*_, the method provides an estimate of what the *μ*_*j *_value should be in the context of the original row *i*.

### Pcons

Pcons values are taken from the CASP website  with the exception of some incorrect values resulting from the bug noted in [3]. Corrected values were obtained from the Pcons authors and the reported performance here reflects the new numbers. Pcons uses LGscore-based structural alignments in place of the LGA minimization employed in this paper. Let *LG*_*j*_(*x*_*i*_) be the distance between the *i*th residue of *S*_*X *_and the *i*th residue of  obtained after structurally superimposing *S*_*X *_and  using the LGscore algorithm [11]. Let *pc*(*x*_*i*_) be the Pcons prediction corresponding to *d*(*x*_*i*_) in Equation 1. Pcons uses the following three equations to produce a prediction.

(3)

(4)

(5)

Note that equation 3 corresponds to the *S*-score for a position in an LGscore alignment.

### Weight learning algorithms

#### Linear perceptron

One way of learning values for *w*_*j *_in Equation 1 is to use Rosenblatt's linear perceptron classifier [17]. This is an online learning algorithm that iteratively updates a weight vector *w *for each training example *x *based on the difference between its actual and predicted values. Pseudo-code for our implementation of this algorithm is shown in the appendix. For each position, the linear perceptron determines the error of each predictor (line 4). Each predictor is then assigned a weight that is inversely related to its error and the vector of these weights (*φ*) is scaled to sum to one (lines 5 and 6). Note that in line 5, the ∑_*j*_*e*_*j*_/*S *factor is used to reduce the difference between lower and higher weights. We found that using this smoothing factor improves results.

The learning rate *α *is updated based on the error of the prediction for each example (*d*(*x*)), as determined using Equation 1. In the case of the unfilled CD6 and CD7 datasets, we use *d*(*x*)/∑_*j*_*w*_*j *_as the prediction. This prevents the sparsity of the set from skewing the values learned for *w*. The vector *φ *becomes the update to *w *(line 8), and *w *is scaled to sum to one after processing each training example (line 9). The final weights are the values of *w *after five iterations over the training data, as a test (results not shown) showed a small difference between the weights from the fourth and fifth iterations. Note that this is a variation on a traditional linear perceptron, in which *w *is updated according to *w *← *w *+ *α*(*real - predicted*)*d*. We use the variation shown in the appendix because it performs better for our problem (results not shown).

#### Support vector regression (SVR)

We used the SVMLight implementation [18] for support vector regression. Default values were used for the tube width and regularization parameters. The details of this regression technique have been described in detail elsewhere [19] and will not be covered here.

#### Standard and constrained regression

We use Matlab for standard regression and for constrained regression in which the weights *w*_*j *_must be positive. We also experimented with a second constrained regression formulation. In this formulation, the weights *w*_*j *_of the predictors must be positive and sum to one. This regression formulation could not learn an appropriate set of weights in the majority of cases, so the results are not included here.

## Authors' contributions

KWD and GK designed the methods, and experimental setup. KWD carried out the implementation of the various methods, and computational experiments. KWD wrote the manuscript under GK's technical supervision and mentoring. Both authors read and approved the final manuscript.

## Appendix

Algorithm 1: Learning Weight Vectors with the linear perceptron algorithm

**Input**: *S*: Number of Predictors.

*m*: Number of Training Samples.

*N*: Number of Training Iterations.

**Output**: *w*: Weight Vector.

1: *w *← 1/*k*

2: **for ***n *= 1 to *N ***do**

3:   **for ***x *= 1 to *m ***do**

4:      *e*_*j *_← |*d*_*j*_(*x*) - *d*_*t*_(*x*) |∀_*j*_

5:      *φ*_*j *_← 1/(*e*_*j *_+ ∑_*j*_*e*_*j*_/*S*) ∀_*j*_

6:      *φ *← *φ*/||*φ*||_1_

7:      *α *← |*d*(*x*) - *d*_*t*_(*x*)|/*m*

8:      *w *← *w *+ *αφ*

9:      *w *← *w*/||*w*||_1_

10:   **end for**

11: **end for**

12: Return *w*
